# A236 BOTH METABOLIC UNHEALTHINESS AND OBESITY ARE REQUIRED TO INCREASE GALLSTONE DISEASE RISK: A LARGE-SCALE CROSS-SECTIONAL STUDY

**DOI:** 10.1093/jcag/gwae059.236

**Published:** 2025-02-10

**Authors:** M Baeg, S Ko

**Affiliations:** Gastroenterology, Jeju National University Hospital, Jeju, Jeju-do, Korea (the Republic of); University of Toronto Temerty Faculty of Medicine, Toronto, ON, Canada

## Abstract

**Background:**

Obesity and metabolic unhealthiness are well-known risk factors of gallstones. However, as obesity and metabolic health are closely related, it is difficult to differentiate its effects.

**Aims:**

The aim of our study was to investigate the relationship between metabolic health status, obesity, and gallstone disease.

**Methods:**

We performed a retrospective cross-sectional study of subjects undergoing health screening at a university hospital. Subjects with abdominal ultrasonography and fasting insulin results were included. Subjects were classified into 4 groups based on metabolic and obesity criteria: metabolically healthy nonobese (MHNO), metabolically healthy obese (MHO), metabolically unhealthy nonobese (MUNO), and metabolically unhealthy obese (MUO). Gallstone disease was defined as those with gallstones on ultrasonography or a history of cholecystectomy. Multivariable analysis was used to identify gallstone disease risk factors with MHNO subjects as reference.

**Results:**

We included 12,052 subjects (6,014 MHNO, 1,662 MHO, 2,211 MUNO, 2,165 MUO). There were 568 subjects with gallstones and 241 with prior cholecystectomy history. The prevalence of gallstone disease was 5.5% in MHNO, 7.1% in MHO, 7.2% in MUNO, and 9.2% in MUO. Gallstone disease risk was significantly increased in the MUO group (OR 1.449, 95% CI 1.198-1.752, P<0.001). but showed only a tendency in the MHO group (OR, 1.219, 95% CI, 0.976–1.522, P=0.080) and was insignificant in the MUNO group (OR, 0.989, 95% CI, 0.808–1.211, P=0.917). Older age significantly increased gallstone disease risk, but alcohol consumption was protective. When metabolic unhealthiness and obesity were analyzed separately, obesity significantly increased gallstone disease risk while metabolic unhealthiness did not.

**Conclusions:**

We demonstrated that gallstone disease risk was significantly increased only in those with both metabolic unhealthiness and obesity.

**Funding Agencies:**

None

